# A Mokken Scale Analysis of the Last Series of the Standard Progressive Matrices (SPM-LS)

**DOI:** 10.3390/jintelligence8020022

**Published:** 2020-05-06

**Authors:** Nils Myszkowski

**Affiliations:** Department of Psychology, Pace University, New York, NY 10038, USA; nmyszkowski@pace.edu

**Keywords:** Mokken scale analysis, non-parametric item response theory, psychometrics, invariant item ordering

## Abstract

Raven’s Standard Progressive Matrices (Raven 1941) is a widely used 60-item long measure of general mental ability. It was recently suggested that, for situations where taking this test is too time consuming, a shorter version, comprised of only the last series of the Standard Progressive Matrices (Myszkowski and Storme 2018) could be used, while preserving satisfactory psychometric properties (Garcia-Garzon et al. 2019; Myszkowski and Storme 2018). In this study, I argue, however, that some psychometric properties have been left aside by previous investigations. As part of this special issue on the reinvestigation of Myszkowski and Storme’s dataset, I propose to use the non-parametric Item Response Theory framework of Mokken Scale Analysis (Mokken 1971, 1997) and its current developments (Sijtsma and van der Ark 2017) to shed new light on the SPM-LS. Extending previous findings, this investigation indicated that the SPM-LS had satisfactory scalability (H=0.469), local independence and reliability (MS=0.841, LCRC=0.874). Further, all item response functions were monotonically increasing, and there was overall evidence for invariant item ordering ($H_{T}=0.475$), supporting the Double Monotonicity Model (Mokken 1997). Item 1, however, appeared problematic in most analyses. I discuss the implications of these results, notably regarding whether to discard item 1, whether the SPM-LS sum scores can confidently be used to order persons, and whether the invariant item ordering of the SPM-LS allows to use a stopping rule to further shorten test administration.

## 1. Introduction

The general factor of intelligence (*g*) is central in the prediction of several outcomes, such as job performance (Ree and Earles 1992; Salgado et al. 2003) and academic achievement (Rohde and Thompson 2007). Its accurate measurement is therefore crucial in multiple contexts, including personnel selection, vocational guidance or academic research in individual differences. However, because of practical constraints, it is desirable in many contexts to reduce test length as much as possible, while maintaining acceptable accuracy.

Raven’s Standard Progressive Matrices (SPM) (Raven 1941) and Advanced Progressive Matrices (APM) (Raven et al. 1962) are widely used—though also criticized (Gignac 2015)—instruments to measure *g*. However, both these tests remain rather long in untimed conditions, with some participants sometimes taking more than 40 min to respond them (Hamel and Schmittmann 2006). Several solutions have been proposed to further reduce test administration time, such as constraining time (Hamel and Schmittmann 2006) and using short versions (Bors and Stokes 1998).

While these solutions have focused on the APM (Hamel and Schmittmann 2006; Bors and Stokes 1998; Myszkowski and Storme 2018) have recently suggested that the last series of the SPM—the SPM-LS—could be a more efficient solution, with only 12 items, while maintaining the progressive aspect characteristic of Raven’s matrices, along with satisfactory psychometric properties. However, the original study (Myszkowski and Storme 2018)—which I propose to extend—has studied the SPM-LS with parametric Item Response Theory (IRT) models, and is largely focused on recovering information from distractor responses using nested logit models (Suh and Bolt 2010; Storme et al. 2019), therefore putting aside important aspects of the test—such as the monotonicity of item responses and invariant item ordering, which I later further discuss. I propose here to bridge these gaps using the framework of Mokken Scale Analysis (MSA) (Mokken 1971, 1982, 1997), a well developed non-parametric item-response theory framework that is particularly appropriate to address them (Sijtsma and van der Ark 2017).

### 1.1. The SPM-LS

While the SPM is heavily studied, the SPM-LS is very recent, and thus has not been the object of many investigations. Currently, it has only been studied in its original investigation (Myszkowski and Storme 2018)—which used binary and nominal IRT models—and as part of this special issue through a further investigation of its dimensionality (Garcia-Garzon et al. 2019). Investigations of the SPM-LS indicated that IRT models could satisfactorily fit test responses (Bürkner 2020; Myszkowski and Storme 2018), and that the test seemed to present adequate reliability/information for abilities ranging from about 2 standard deviations below the mean—or 3 if recovering information from distractors—to 1.5 to 2 standard deviations above the mean (Myszkowski and Storme 2018), in a sample of undergraduate students, suggesting that it could be more appropriate in terms of difficulty for the general population than for post-secondary students. In addition, Garcia-Garzon et al. (2019) notably studied in this special issue the dimensionality of the SPM-LS using a variety of methods—Exploratory Graph Analysis (EGA), bifactor Exploratory Factor Analysis (EFA) and Confirmatory Factor Analysis (CFA). Overall, the psychometric qualities of the SPM-LS so far appeared satisfactory for use by researchers and practitioners, but some characteristics have not been studied, for which Mokken Scale Analysis is a particularly appropriate framework.

### 1.2. Mokken Scale Analysis

Since its inception (Mokken 1971), Mokken Scale Analysis has been the object of several methodological developments, notably discussing how to evaluate the properties of instruments evaluated with MSA (Van der Ark 2012), best practices in MSA (Sijtsma and van der Ark 2017) and the active development of a package (Van der Ark 2007) for the statistical programming language R. While it is largely and more thoroughly described elsewhere (Mokken and Lewis 1982; Mokken 1997; Van der Ark 2007; Sijtsma and van der Ark 2017; Sijtsma 1998), I could briefly describe Mokken Scale Analysis (MSA) (Mokken and Lewis 1982; Mokken 1997) as a non-parametric IRT framework, which, for dichotomous responses, represents the probability of succeeding an item *j* as a function of an person *i*’s latent ability—$\theta_{i}$. Unlike the Rasch model (Rasch 1993) and, more broadly, unlike binary logistic and normal ogive models—which are said to be parametric IRT models (Mokken and Lewis 1982)—MSA does not represent the relation between latent ability and item responses using item parameters, but using an item-response function only defined as monotonically increasing (Mokken and Lewis 1982).

### 1.3. The Benefits of Mokken Scale Analysis

Because they do not require response functions to have a specific shape, Mokken’s models are less constrained than (notably) Rasch models (Meijer et al. 1990), which implies that some items that are not well fitted by Rasch models may still be scalable with MSA, because their response function may be monotonic without necessarily having a logistic/normal ogive shape. While MSA does not allow certain applications otherwise permitted by Rasch modeling, like test equating or computer adaptive testing, (Meijer et al. 1990, p. 297) note that, “for many testing applications, it often suffices to know the order of persons on an attribute”. Therefore, Mokken scaling is attractive for the reason that it focuses mainly on a test’s capacity to order persons, while allowing for more items to fulfill its requirements than Rasch models do allow. In the context of the SPM-LS, this is particularly interesting, especially as Myszkowski and Storme (2018) had to use highly parametrized models to achieve an acceptable fit, with 3- and 4-parameter models fitting much better than notably the Rasch 1-parameter model—in this special issue, Bürkner (2020) makes a similar conclusion using Bayesian IRT. Instead of increasing the number of parameters to better fit item responses—and risking overfitting and thus compromising reproducibility—Mokken scaling proposes to retain fewer (but fundamental) uses of a test: Ordering persons (for both MSA models) and items (for the Double Monotonicity model only).

#### 1.3.1. The Monotone Homogeneity and Double Monotonicity Models

For dichotomous items, Mokken (1997) defined two item-response models: The Monotone Homogeneity Model (MHM) and the Double Monotonicity Model (DMM). Both the Monotone Homogeneity Model and the Double Monotonicity Model assume the monotonicity of item response functions. However, the two models differ in that only the Double Monotonicity Model assumes that item response functions do not intersect—an assumption usually referred to as invariant item ordering.

Before focusing on these two assumptions central to MSA, as well as their consequences in the context of the SPM-LS, it is important to note that both models also assume unidimensionality, meaning that they both assume that the same latent attribute $\theta_{i}$ explains the items scores—therefore also assuming local independence (Sijtsma et al. 2011). While MSA offers procedures (also used in this study) to investigate this assumption, they would probably not justify a new study, because the dimensionality of the SPM-LS has been, on this very dataset, investigated with a plethora of psychometric methods (Myszkowski and Storme 2018; Garcia-Garzon et al. 2019). I will therefore mainly focus here on the *incremental* value of using Mokken Scale Analysis in addition to these previously used approaches.

#### 1.3.2. Monotonicity of Item Response Functions

An important feature of MSA is that it allows to study monotonicity, where parametric Item-Response Theory and traditional (linear) factor analysis models generally leave this assumption untested. Indeed, although parametric item response models for binary responses are (in general) monotonous, a misfitting item does not necessarily indicate that the item response is non-monotonic (Meijer et al. 1990). Therefore, because it has only been studied with parametric response models, the monotonicity of the SPM-LS has remained untested so far. This characteristic is manifested, in pass-fail (binary) tests like the SPM-LS, by item response functions that are monotonically increasing. This means that the probability to succeed on an item monotonically increases with the examinee’s ability. In contrast with parametric IRT, the framework of Mokken Scale Analysis offers methods to investigate this property and specifically identify its violations (Van der Ark 2007). I therefore propose, in the present study, to use this framework to bridge that gap in the study of the test.

As a consequence, this study is therefore the first to study the monotonicity of the SPM-LS, which, as previously noted (Van der Ark 2007), is not only relevant to Mokken scaling, but also relevant to any model that formulates this assumption, such as parametric Item-Response Theory models and traditional factor analysis. It is an essential psychometric property of a test, because it is the property that implies that higher scores imply higher abilities (for all items, at any ability level), and thus that scores can be used to infer person ordering (Van der Ark 2007).

#### 1.3.3. Invariant Item Ordering

While both the Monotone Homogeneity Model and the Double Monotonicity Model assume unidimensionality and monotonicity of the response functions, only the Double Monotonicity Model assumes that the ordering of the items (based on their difficulty) is the same for all examinees (Mokken 1997; Sijtsma and van der Ark 2017; Sijtsma et al. 2011). In other words, this property, referred to as Invariant Item Ordering (IIO), assumes that, for any given item pair, the easier item has a higher probability of being succeeded than the more difficult one at any ability level. This manifests itself graphically by the item response functions of the two items not intersecting.

As was previously noted (Sijtsma et al. 2011; Sijtsma and van der Ark 2017), this property is an important feature of a test, as it “greatly facilitates the interpretation of test scores” (Sijtsma and van der Ark 2017, p. 143), and is “both omnipresent and implicit in the application of many tests, questionnaires, and inventories” (Ligtvoet et al. 2010, p. 593). Indeed, a stronger IIO implies that two persons with the same total score are more likely to have succeeded the same items, and that an examinee with a higher total score than another examinee is more likely to have answered correctly the same items, and one or several more difficult items. Therefore, invariant item ordering lends more meaning to person comparisons based on total scores.

In addition, IIO is especially relevant for the SPM-LS, because its items substantially vary in difficulty and are presented by increasing difficulty. A stronger IIO implies that, if an examinee fails an item, there is an increased probability that the examinee will fail the next (more difficult) one. Therefore, a stronger IIO would suggest that we can envision stopping the test administration after one or several items have been failed (Sijtsma and Meijer 1992). This would presents practical advantages, notably for shortening test administration.

## 2. Materials and Methods

### 2.1. Participants

Per the topic of this special issue, I re-analyzed the publicly available dataset from Myszkowski and Storme (2018) study. The original study presented various parametric IRT analyses performed on a dataset comprised of 499 students (214 males and 285 females) aged between 19 and 24. Because I directly reanalysed this dataset, I point to the original article for more details on data collection and sample characteristics.

One thing to note that is specific to this paper is that the sample size is both similar to the one used in Sijtsma and van des Ark’s tutorial on Mokken scale analysis (Sijtsma and van der Ark 2017) and, more importantly, in accordance with the sample size recommendations provided by Straat et al. (2014). They show (p. 817) that a sample size of around 500 is largely sufficient for an accurate analysis with scalability coefficients $H_{j}$ of 0.42 (or higher)—in the results, I present scalability coefficients, and show that the scalability of the scale meets that requirement.

### 2.2. Instrument

The Last Series of the Standard Progressive Matrices, or SPM-LS (Myszkowski and Storme 2018), was built from the original Standard Progressive Matrices (Raven 1941), a very popular and extensively researched test of non-verbal logical reasoning, which is also frequently used as a brief measure of *g*, the general factor of intelligence. As its name indicates, it consists of the last—and thus most difficult—series of the original SPM, but used as a standalone test (without examinees taking previously the other series). It is composed of 12 items of theoretically increasing difficulty. Each item consists of an incomplete 3-by-3 matrix, with the last element of the matrix being missing. The examinee is to identify, among eight options—seven distractors and one correct response—the missing matrix element.

Research shows that *g* is far from being extensively, nor purely captured by the SPM (Gignac 2015; Carpenter et al. 1990), and this is certainly even more true of SPM-LS, since it is a shortened version. Nevertheless, the SPM, and a fortiori the SPM-LS, present the advantage of being short measures, with overall satisfactory reliability. In particular, the SPM-LS, in its original investigation on this dataset (Myszkowski and Storme 2018), presented encouraging evidence of reliability, with observed reliabilities based on IRT modeling that ranged from 0.78 to 0.84 depending on the IRT model used, and a Cronbach’s α of 0.92.

As unidimensionality is an assumption of Mokken Scale Analysis (Van der Ark 2007), it is also important to note that the SPM-LS investigations indicated that the test is essentially unidimensional, with a McDonald’s coefficient $\omega_{h}$ of 0.86 (Myszkowski and Storme 2018) and satisfactory fit of unidimensional models (Myszkowski and Storme 2018). Garcia-Garzon et al. (2019) explorations also supported unidimensionality, in spite of a nuisance factor specific to the last six items.

### 2.3. Analysis

Because Sijtsma and van der Ark’s tutorial on Mokken scale analysis (Sijtsma and van der Ark 2017) presents the advantages of presenting the current state of the art of Mokken scale analysis and of laying out clearly the different steps to take in order to perform a Mokken scale analysis, I followed the different steps provided in the tutorial. All analyses were computed using the same team’s regularly updated and comprehensive R package mokken (Van der Ark 2007, 2012; Sijtsma et al. 2011) (version 2.8.11).

A reason for the popularity of Mokken scaling is the availability of an automatic procedure to select a set (or several sets) of scalable items, a procedure generally referred to as the Automated Item Selection Procedure (AISP), which aims at maximizing scalability. In addition, Straat et al. (2016) also recently suggested a item selection procedure which aims to maximize local independence. Likewise, a stepwise selection procedure aiming at maximizing invariant item ordering has been proposed (Ligtvoet et al. 2010). Still, it was decided here that the primary objective of the present study would be to investigate the SPM-LS as an a priori scale, meaning that the main objective was to investigate its qualities using Mokken Scale Analysis, not to carve a revised instrument out of it. This decision was motivated by the fact that the SPM-LS is already a very short measure (12 items), and also because, in the SPM-LS, the very process of solving items is—at least theoretically—used to help the examinee learn the rule(s) used in subsequent items (Myszkowski and Storme 2018). Therefore, even if an item were to present poor qualities (e.g., weak scalability), it might still be useful as a training for the other items, and thus it may still be preferable or conservative to keep it.

#### 2.3.1. Data Preparation

The dataset analyzed did not present any missing data nor impossible responses. Sijtsma and van der Ark (2017) recommend, as a preliminary step to Mokken Scale Analysis, to filter out cases whose responses are dubious, and they suggest doing so using the count of Guttman errors. I proceeded to count the number of Guttman errors $G_{+}$ per case, computed with the package function check.errors() of the mokken package. There were a total of 2021 Guttman errors, indicating that the items did not constitute a Guttman scale.

**Figure 1:**
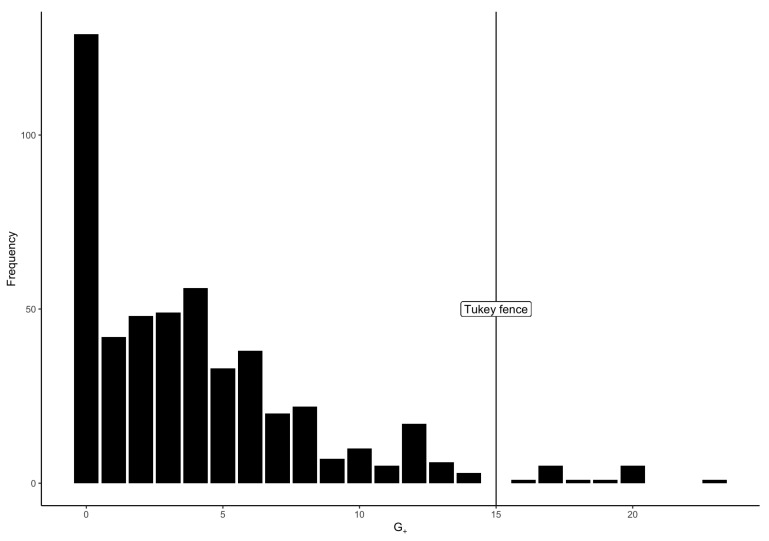
Histogram of the count of Guttman errors ($G_{+}$), with Tukey fence (3rd quartile +1.5×IQR) used as a threshold for outlier detection.

As Sijtsma and van der Ark (2017) suggested, I identified as dubious cases—and consequently removed—the cases for which $G_{+}$ indices were beyond the upper Tukey fence of the distribution of $G_{+}$ indices. This corresponded to cases with more than 15 Guttman errors, and resulted in the elimination of 14 cases (1.17% cases) with suspicious item-score patterns. The frequency histogram of $G_{+}$ indices, with the Tukey fence, is presented in Figure 1.

#### 2.3.2. Scalability

As recommended by Sijtsma and van der Ark (2017), I investigated the scalability of the complete SPM-LS by computing $H_{jk}$ (scalability coefficients for item pairs), $H_{j}$ (scalability coefficients for items) and *H* (total scalability coefficient of the scale). I used the rules of thumb originally proposed by Mokken (1997) and currently suggested by Sijtsma and van der Ark (2017), which are H<0.3 for insufficient scalability, 0.3≤H<0.4 for weak scalability, 0.4≤H<0.5 for medium scalability and H≥5 for strong scalability. Since the Monotone Homogeneity Model implies that $H_{jk}$ and $H_{j}$ are all positive (and ideally as close to 1 as possible), I searched for negative values (or values close to 0) as violations of the monotonicity (Sijtsma and van der Ark 2017).

#### 2.3.3. Local Independence

Local independence is an assumption of both the monotone homogeneity model and the double monotonicity item. Local independence implies that item scores are independent for a given ability level θ. As suggested in Sijtsma and van der Ark (2017)’s tutorial, I used the procedure proposed by Straat et al. (2016) to study local dependencies in the SPM-LS. They suggest the computation of three series of indices: $W_{1}$, $W_{2}$ and $W_{3}$. While the computation of these indices is further explained in the original article, we can note that high $W_{1}$, $W_{2}$ and $W_{3}$ values indicate local dependencies. High $W_{1}$ values indicate that an item pair is likely positively locally dependent. An item with a high $W_{2}$ is likely to be positively locally dependent with another item. High $W_{3}$ indicate that an item pair is likely negatively locally dependent. Again here, and as Straat et al. (2016) suggested, a Tukey fence was used to detect problematic items.

#### 2.3.4. Monotonicity

As recommended by Sijtsma and van der Ark (2017), I studied monotonicity by plotting item response functions, using a non-parametric regression of each item scores on “rest scores” (the total scores on the other items) (Junker and Sijtsma 2000). Following the defaults of the check.monotonicity() function of the mokken package (Van der Ark 2007), the rest scores were grouped using a minimum size of N/5 (meaning groups of 100 cases at least). The identified violations were then significance tested (Van der Ark 2007; Molenaar and Sijtsma 2000).

As an alternative to testing monotonicity violations, it has been been recently proposed that positive evidence for monotonicity can be gathered through Bayes factors (Tijmstra et al. 2015; Tijmstra and Bolsinova 2019). Based on a suggestion by a reviewer that I use this procedure, I contacted the first author of these papers, who provided code to implement it. The procedure is discussed in more details in the original paper (Tijmstra et al. 2015), but, in short, it consists in evaluating the relative amount of support from the data for (strict) manifest monotonicity—denoted hypothesis $H_{MM}$—against the competing hypothesis that there is at least one manifest non-monotonicity—denoted hypothesis $H_{NM}$—and against the competing hypothesis of essential monotonicity—denoted $H_{EM}$, defined as a form of monotonicity that allows for non-monotonicities between adjacent manifest scores. Bayes Factors $BF_{MM,NM}$ and $BF_{MM,EM}$ were estimated through Gibbs sampling, and used to indicate support for $H_{MM}$ in contrast with $H_{NM}$ and $H_{EM}$ respectively. Values above 1 indicate more support for $H_{MM}$ than for the competing hypothesis. 20,000 iterations were used as burn-in and discarded, and 100,000 iterations were subsequently used to estimate the Bayes Factors—which is more conservative than initially suggested (Tijmstra et al. 2015).

#### 2.3.5. Invariant Item Ordering

Even though Invariant Item Ordering (IIO) is only an assumption of the Double Monotonicity Model for binary items (Mokken 1997)—not of the the Monotone Homogeneity Model—I studied IIO because of its benefits for score interpretability and the possibility to stop the examination after failed items. As Sijtsma and van der Ark (2017) suggested, overall IIO was assessed with the coefficient $H_{T}$. Like for the *H* scalability coefficients, and as suggested by Ligtvoet et al. (2010), I used thresholds of $H_{T}<0.3$ for insufficient IIO (Sijtsma and Meijer 1992), $0.3\leq H_{T}<0.4$ for weak IIO, $0.4\leq H_{T}<0.5$ for medium IIO and $H_{T}\geq 5$ for strong IIO. I also graphically compared the item response functions of pairs of items that significantly intersect.

#### 2.3.6. Reliability

Cronbach’s α and empirical reliability from parametric IRT models have been previously reported and discussed as satisfactory in the same dataset (Myszkowski and Storme 2018). Here, to investigate reliability, as recommended in the context of MSA (Sijtsma and van der Ark 2017), I used the Molenaar-Sijtsma (MS) reliability estimate (Sijtsma and Molenaar 1987), which assumes the Double Monotonicity Model. In addition, I reported the Latent Class Reliability Coefficient (LCRC) (van der Ark et al. 2011), which is more robust to violations of the Double Monotonicity Model.

## 3. Results

### 3.1. Scalability

The SPM-LS had medium scalability, with an *H* coefficient of 0.469 (SE=0.021). The scalability of the item pairs $H_{jk}$ is reported in Table 1, along with the scalability of the items $H_{j}$. All item pairs and item scalability coefficients were positive, giving support to the monotone homogeneity model. However, it can be noted that the first item had a substantially lower scalability ($H_{j}=0.265$) than the other items ($H_{j}$ ranging from 0.401 to 0.602), and that the total scalability would be strong (H=0.516) without this item.

### 3.2. Local Independence

The $W_{1}$, $W_{2}$ and $W_{3}$ indices for local dependencies detection are presented in Table 2. While $W_{2}$ indices did not suggest that any item were likely in a positive locally dependent pair, $W_{1}$ indices identified 3 positive local dependencies, between Item 4 and 11, 5 and 11, and 5 and 12. $W_{3}$ indices suggested a negative local dependency between item 1 and 9.

### 3.3. Monotonicity

**Figure 2:**
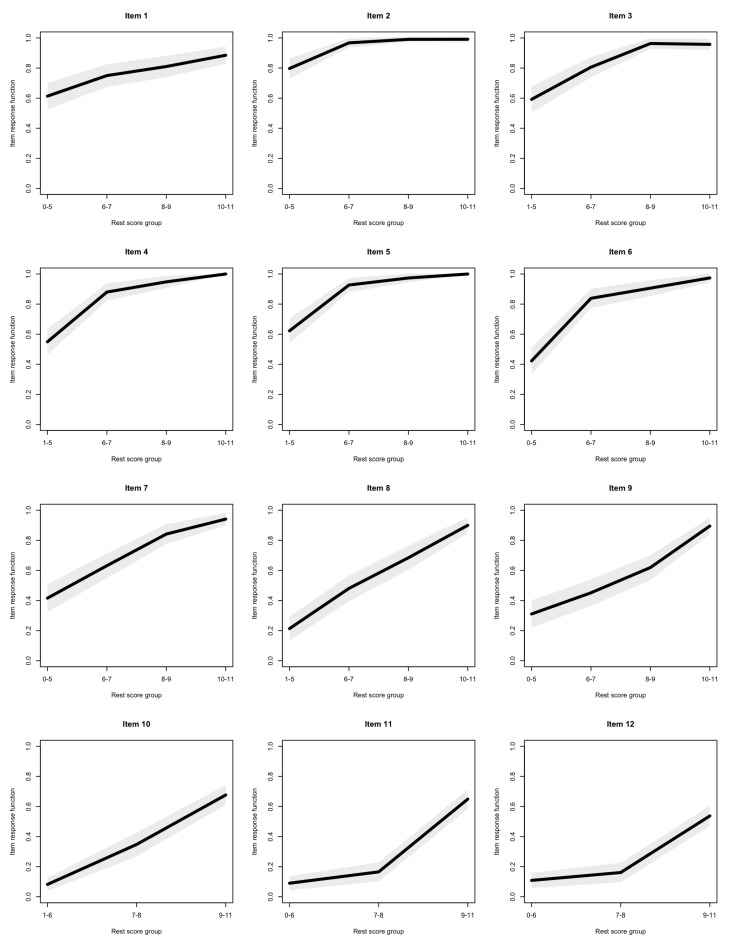
Item response functions of the last series of the Standard Progressive Matrices (SPM-LS) items (with 95% confidence intervals).

The item response functions of all items are presented in Figure 2. Only one violation of monotonicity was observed, for item 3—the response function of this item can be seen as slightly decreasing between rest scores 8–9 and 10–11. This violation was, however, non significant.

The Bayes Factors used to compare the relative support for monotonicity against non-monotonicity and essential monotonicity are reported in Table 3. Overall, monotonicity was supported for all items against its complement—although the support was much weaker for the last two items—with Bayes Factors ranging from 1.64 to 818,417.9. The data tended to support (strict) monotonicity against essential monotonicity, with, however, limited support, and with the exception of 12, which had a Bayes Factor slightly smaller than 1.

### 3.4. Invariant Item Ordering

**Figure 3:**
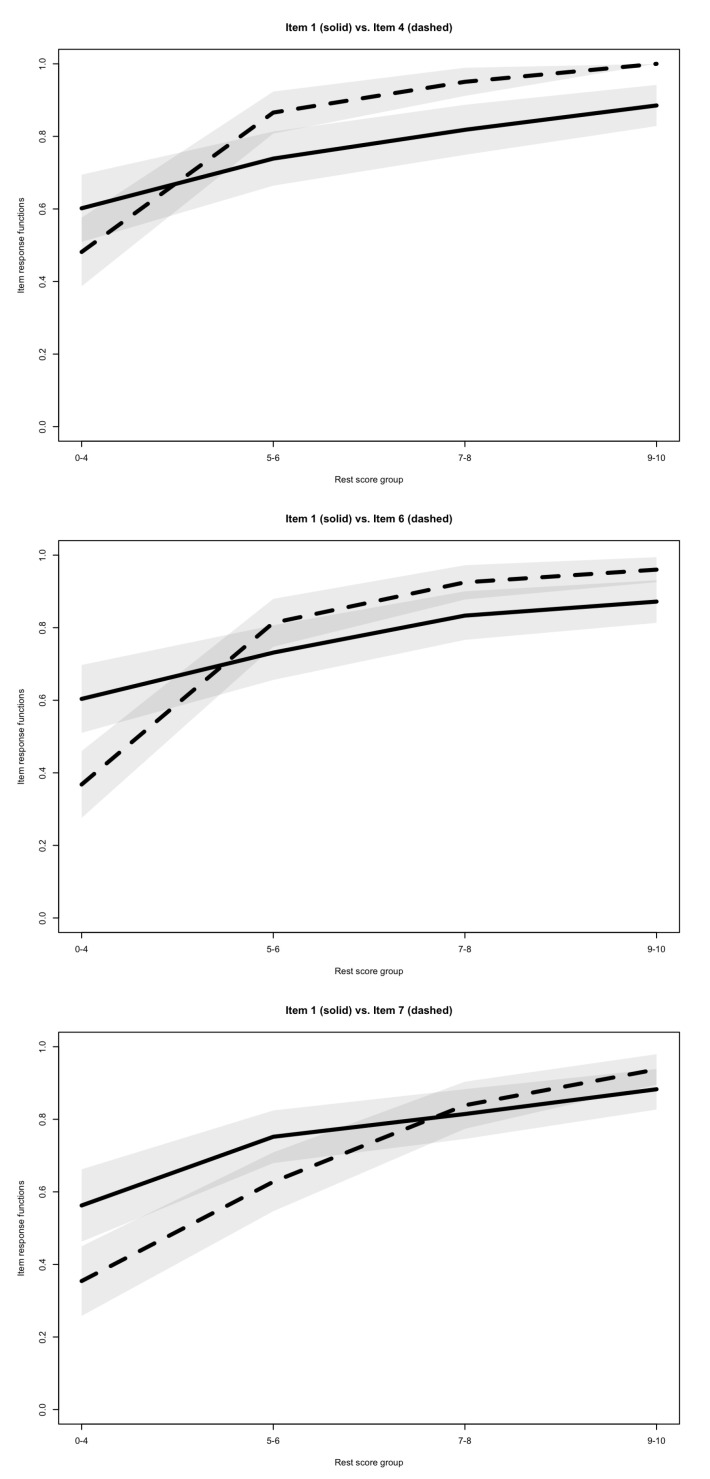
Item response functions (with 95% confidence intervals) of significantly intersecting item pairs.

The observed invariant item ordering was medium but close to strong, with a $H_{T}$ coefficient of 0.475, overall supporting IIO, and therefore, in combination with the previous analyses, supporting the Double Monotonicity Model. Only 3 significant violations of IIO were observed, involving item 1 with items 4, 6 and 7. The item response functions for item pairs with significant intersections are presented in Figure 3^1^. Because all three violations involved item 1, I computed $H_{T}$ again without it, and found that the IIO would in this case be strong ($H_{T}=0.520$).

### 3.5. Reliability

The MS reliability estimate was 0.836, and the LCRC reliability estimate was 0.876, both indicating, like previously found using other estimates (Myszkowski and Storme 2018), that the SPM-LS had satisfactory reliability. The item-rest correlations ranged between 0.285 and 0.563, item 1 having a notably lower item-rest correlation that the other items. However, the reliability indices were similar without this item (MS=0.841, LCRC=0.874).

## 4. Discussion

While the SPM-LS has already been investigated using a variety of methods in this very dataset—including parametric IRT, Bayesian IRT, factor analysis, and exploratory graph analysis (Myszkowski and Storme 2018; Garcia-Garzon et al. 2019; Bürkner 2020)—the current study proposes the first investigation of this instrument using non-parametric IRT, and more specifically Mokken Scale Analysis (Mokken 1971; Mokken and Lewis 1982). This framework allowed to study several psychometric properties, permitting to both confirm the previous encouraging results on the SPM-LS—on dimensionality, local independence and reliability—and to investigate new properties—monotonicity and invariant item ordering.

### 4.1. Conclusions on the SPM-LS

Overall, the SPM-LS showed robust psychometric qualities in this study. More specifically, it was found to have satisfactory monotonicity, scalability, local independence (with only a few local dependencies), invariant item ordering (with only a few significant violations) and reliability. This is an overall satisfactory set of results, which would lead us to encourage the use of this instrument.

The main new elements regarding the investigation of this scale were the support for monotonicity—the item response functions were overall monotonically increasing—and invariant item ordering—the item response functions overall did not intersect, giving, along with unidimensionality and local independence, support for the Double Monotonicity Model. The fact that this model was overall supported is interesting, as it presents several advantages for the use of the SPM-LS in practice (Ligtvoet et al. 2010). First, the monotonicity of item responses suggests that, even though Rasch 1-parameter (and to a lesser extent, 2-parameter) models did not fit well this dataset (Myszkowski and Storme 2018; Bürkner 2020), there is support for the SPM-LS sum scores being able to order persons based on their ability. In addition, it is very clear that each series of Raven’s matrices were originally conceptualized as having a cumulative structure, with examinees responding items gradually increasing in difficulty by the stacking of logical rules to decipher and apply: Empirical support for invariant item ordering supports such a hypothetical functioning of the test. Test editors and practitioners generally assume, that, because an item A has a higher success rate than another item B, then item A is necessarily easier than B for all examinees, and they often use a test as though this assumption were true, without empirically testing it (Ligtvoet et al. 2010): The current study provides evidence that it is empirically justified to make such interpretations from the SPM-LS.

It was notable, through this investigation, that the issues encountered tended to involve item 1. More specifically, item 1 was the item with the smallest scalability (based on $H_{j}$ coefficients), the only one with an outlying negative local dependency (based on $W_{3}$ coefficients), was involved in all three significant violations of invariant item ordering, and had the lowest item-rest correlation. While it appears tempting to remove this item, I would recommend to at least maintain it as a training item (meaning, having participants take it but not necessarily including it in the scoring). This is because (1) the presence of this item is still probably important for the examinees to learn the base rule used throughout the series, and (2) the plots suggest that this items’ response function is still monotonous, and its intersections with the item response functions of items 4, 6 and 7 appear somewhat minimal, as the confidence intervals overlap for most ability levels. The current study suggests that practitioners and/or future researchers using the SPM-LS use the full instrument, even though they may question and study their own dataset to decide on whether to use item 1 in the scoring or not.

### 4.2. Limitations

While this investigation presents satisfactory findings regarding the psychometric qualities of the SPM-LS, the different indices observed were not perfect, and notably, the scalability of the scale was only medium (Mokken 1997; Sijtsma and van der Ark 2017), suggesting that the instrument can further be improved. I noted earlier that it would be categorized as strong if item 1 were excluded from the scoring but, albeit strong, it would be still just above the strong threshold. In addition, excluding item 1 from scoring remains a post-hoc suggestion, made after seeing each items’ scalability. It would therefore call for further investigations using a new sample.

Mokken Scale Analysis investigates aspects of psychometric instruments that are different from more usual sets of analyses (notably of the factor analytic or Rasch tradition)—especially the investigation of monotonicity and invariant item ordering—but this study also suffers from some limitations of this specific framework. For example, it does not provide a way to study or recover information from distractor responses like other approaches—such as nested logit models (Suh and Bolt 2010), the nominal response model (Bock 1997) or the multiple-choice model (Thissen and Steinberg 1984)—which are an important aspect of this specific test (Myszkowski and Storme 2018). Related to this, MSA certainly allows to graphically study item responses, but, because it is non-parametric, it does not produce item parameters that can be interpreted. This is a limiting factor in this context, because previous results (Myszkowski and Storme 2018; Bürkner 2020) suggest that phenomena like guessing—which is unaccounted for in MSA, apart from potentially appearing in item response functions—are relevant for this test. Another limitation of MSA is that it does not provide a way to investigate conditional reliability, and therefore does not allow to, for example, diagnose if an instrument provides reliable ability estimates across a wide range of ability levels. This is particularly a problem in the case of the SPM-LS, because the fact that it only includes one series of the original SPM implies that the range of abilities that are reliably measured may be limited (Myszkowski and Storme 2018). Finally, other advanced uses of Rasch modeling, such as computer-adaptive testing and test equating, are also impossible with Mokken scaling (Meijer et al. 1990).

### 4.3. Future Directions

Support for the Double Monotonicity Model, because of invariant item ordering, indicates that, for an item A of lower difficulty than an item B, an examinee who fails item A is predicted to also fail item B (and all items that are more difficult). Thus, if one orders items from the easiest to the most difficult, as is done with the SPM-LS, then it is conceivable to have examinees stop the test after a number of failures. This is because they are likely to then fail all future items. As a supplementary analysis, in this dataset, I computed the correlations between the full scores of examinees (using all item scores) and the scores they would have received, had they been stopped after a number of consecutive failures. I found that stopping the test after only one failure provided scores that were strongly (but far from perfectly) correlated with full scores—r(483)=0.735, p<0.001—while stopping the test after two consecutive items failed would preserve scores nearly perfectly—r(483)=0.999, p<0.001. Based on this, I would suggest that stopping the administration after 2 consecutively failed items could lead to gains of administration time without any substantial loss of information about an examinee’s ability. I recommend that future studies further examine this possibility, though the present study already gives quite a strong support for such a use.

Finally, while the psychometric investigation of an instrument can take many shapes, the current study demonstrates how Mokken Scale Analysis can provide insightful information about an instrument, even when that instrument has already been studied in the same dataset with multiple popular and less popular methods (Myszkowski and Storme 2018; Bürkner 2020; Garcia-Garzon et al. 2019). Besides replicating the present study in other samples and in other conditions—which is certainly called for—I suggest that future studies investigate the SPM-LS using other non-parametric IRT models—for example, spline IRT models (Winsberg et al. 1984)—to better understand its functioning.

## Figures and Tables

**Table 1:** Scalability coefficients of the item pairs ($H_{j}k$) and items ($H_{j}$).

Index	Item	1	2	3	4	5	6	7	8	9	10	11	12
$H_{jk}$	1												
	2	0.616											
	3	0.331	0.535										
	4	0.248	0.613	0.286									
	5	0.294	0.493	0.421	0.772								
	6	0.272	0.511	0.509	0.520	0.675							
	7	0.122	0.544	0.295	0.471	0.575	0.362						
	8	0.263	0.647	0.442	0.636	0.701	0.547	0.429					
	9	0.095	0.393	0.460	0.441	0.481	0.416	0.427	0.378				
	10	0.327	0.709	0.799	0.938	0.921	0.743	0.526	0.449	0.403			
	11	0.399	0.664	0.569	0.822	0.774	0.677	0.595	0.506	0.522	0.467		
	12	0.267	0.717	0.253	0.839	0.847	0.576	0.613	0.602	0.462	0.400	0.449	
$H_{j}$		0.265	0.568	0.426	0.545	0.602	0.499	0.422	0.476	0.401	0.529	0.536	0.500

**Table 2:** $W_{1}$, $W_{2}$ and $W_{3}$ indices of the SPM-LS (flagged values in bold face).

Index	Item	1	2	3	4	5	6	7	8	9	10	11	12
$W_{1}$	1		3.044	3.621	3.842	3.852	4.714	2.897	3.963	1.857	2.956	1.955	1.556
	2	0.508		1.865	3.742	3.196	1.908	1.497	2.806	0.661	1.932	0.349	0.309
	3	1.073	2.381		2.512	3.065	2.503	1.324	2.432	1.449	3.655	0.909	0.634
	4	0.036	2.128	0.503		3.039	1.018	0.468	1.772	0.095	2.959	0.019	0.162
	5	0.072	1.844	0.430	3.299		1.043	0.591	2.739	0.181	2.915	0.021	0.037
	6	0.352	1.996	0.389	1.668	2.098		0.392	1.237	0.159	2.578	0.101	0.704
	7	0.766	2.287	1.529	2.730	3.621	1.666		1.523	0.779	2.265	0.282	0.310
	8	0.136	1.450	0.445	2.325	2.537	0.721	0.775		0.502	0.605	0.045	1.769
	9	0.483	3.996	2.731	3.375	3.486	2.376	2.507	3.225		2.176	1.241	0.873
	10	0.077	3.838	1.735	3.742	3.316	1.245	0.893	0.317	0.326		0.448	0.107
	11	0.425	5.611	1.779	**8.765**	**8.429**	2.972	1.813	0.854	1.451	0.994		0.274
	12	1.137	5.129	1.017	5.455	**7.380**	3.288	2.525	2.370	2.441	2.234	2.089	
$W_{2}$		49.281	38.952	42.890	30.910	27.740	39.323	44.227	33.246	44.265	28.471	35.393	33.611
$W_{3}$	1										
	2	3.116											
	3	3.487	2.708										
	4	4.338	3.408	5.276									
	5	3.826	3.321	3.457	0.297								
	6	4.534	5.199	1.561	2.944	2.022							
	7	6.683	3.861	5.579	2.820	1.616	5.398						
	8	3.869	3.653	4.941	2.959	2.919	3.122	2.187					
	9	**7.181**	5.033	3.288	4.118	4.116	5.143	4.575	1.376				
	10	4.405	3.469	2.120	0.990	1.626	2.111	3.628	3.555	2.584			
	11	3.540	2.756	4.269	2.037	2.424	3.074	5.086	3.468	3.870	1.604		
	12	4.303	2.428	6.204	1.723	2.116	4.215	2.797	1.198	2.982	2.378	3.267	

**Table 3:** Bayes Factor for the relative support of monotonicity against its complement ($BF_{MM,NM}$) and against essential monotonicity ($BF_{MM,EM}$).

Item	${BF}_{MM,NM}$	${BF}_{MM,EM}$
1	825.15	1.44
2	34,666.90	3.49
3	57,682.66	3.89
4	871,824.00	8.26
5	89,668.37	4.20
6	95.22	1.15
7	9594.47	4.13
8	818,417.90	6.40
9	12.08	2.58
10	50,455.13	3.80
11	1.98	4.31
12	1.64	0.81

## Footnotes

1. For Figure 3, the plotting function of the mokken package was modified in order for all rest score groups on the *x*-axis to be consistent. In addition, it can be noted that the three plots involve item 1, but that its item response function appears slightly different in the three plots. The reason for this is that the rest score is computed in each plot using all items but the two items involved in the comparison. Since the item pair is different in each plot, the rest score group is therefore different, leading to slightly different response functions for the same item.
